# Antiosteoporotic Activity of *Dioscorea alata* L. cv. Phyto through Driving Mesenchymal Stem Cells Differentiation for Bone Formation

**DOI:** 10.1155/2011/712892

**Published:** 2011-06-29

**Authors:** Kang-Yung Peng, Lin-Yea Horng, Hui-Ching Sung, Hui-Chuan Huang, Rong-Tsun Wu

**Affiliations:** ^1^Institute of Biopharmaceutical Science, School of Pharmaceutical Science, National Yang-Ming University, Taipei City 112, Taiwan; ^2^Research Center for Drug Discovery, National Yang-Ming University, Taipei City 112, Taiwan

## Abstract

The aim of this study was to evaluate the effect of an ethanol extract of the rhizomes of *Dioscorea alata* L. cv. Phyto, Dispo85E, on bone formation and to investigate the mechanisms involved. Our results showed that Dispo85E increased the activity of alkaline phosphatase (ALP) and bone nodule formation in primary bone marrow cultures. In addition, Dispo85E stimulated pluripotent C3H10T1/2 stem cells to differentiate into osteoblasts rather than adipocytes. Our *in vivo* data indicated that Dispo85E promotes osteoblastogenesis by increasing ALP activity and bone nodule formation in both intact and ovariectomized (OVX) mice. Microcomputed tomography (*μ*CT) analysis also showed that Dispo85E ameliorates the deterioration of trabecular bone mineral density (tBMD), trabecular bone volume/total volume (BV/TV), and trabecular bone number (Tb.N) in OVX mice. Our results suggested that Dispo85E is a botanical drug with a novel mechanism that drives the lineage-specific differentiation of bone marrow stromal cells and is a candidate drug for osteoporosis therapy.

## 1. Introduction

Osteoporosis is a prevalent bone disease that is defined by a low bone mass and an increased risk of fractures [1]. Adequate regulation of bone remodeling in adulthood is essential to maintain a healthy bone mass [2]. However, an imbalance between bone formation and bone resorption in pathological conditions or during the aging process can result in conditions that lead to osteoporosis [3]. In the past, the process of bone remodeling was considered to be regulated by two major cell types: bone-forming osteoblasts and bone-resorbing osteoclasts [4]. Currently available agents used to treat osteoporosis include estrogen, raloxifene, the bisphosphonates (e.g., alendronate and risedronate, etc.), and calcitonin. Their mechanisms are based on the inhibition of osteoclastic bone resorption to prevent further bone loss [5]. However, many osteoporotic patients already had lost a substantial amount of bone mass before diagnosis [6]. Furthermore, many side effects of antiresorptive agents have been reported, causing many patients to discontinue their use [7–9].

Drugs with anabolic effects have received much recent attention for osteoporosis therapy. These pharmacologic agents can ultimately stimulate new bone formation, enhance bone density, reduce bone fracture, and promote bone health. Currently, there is only one available anabolic agent on the market approved for the treatment of osteoporosis, recombinant human parathyroid hormone (hPTH). However, hPTH is limited for use in cases of severe osteoporosis [10], and the clinical treatment period is limited to 18 months [11]. Consequently, it is necessary to develop other potential anabolic agents with fewer undesirable side effects to prevent or reverse osteoporosis.

The Chinese yam (*Dioscorea*) has been widely used as a herbal medicine in China for more than 2000 years. Ancient Chinese medicinal books or folk remedies have provided evidence that *Dioscorea* might have effects in regulating bone metabolism. Therefore, we initiated a project to evaluate the effects of an ethanol extract of rhizomes of *Dioscorea alata* L. cv. Phyto, Dispo85E, on bone formation and to delineate the mechanisms involved. In our study, we found that Dispo85E promoted bone formation by inducing mesenchymal stem cells (MSCs) differentiation into osteoblasts rather than adipocytes and that it also possessed antiosteoporotic activity *in vivo*.

## 2. Materials and Methods

### 2.1. Animals

Specific pathogen-free (SPF) C57BL/6 female mice, 8 to 10 weeks of age, were obtained from the National Laboratory Animal Center (Taipei, Taiwan). Animals were housed at a constant temperature and fed with laboratory chow (PMI, Brentwood, Mo, USA) and water *ad libitum*. The protocol of the experiments was approved by the Animal Research Committee of National Yang-Ming University (Guide for Animal Experiments, National Yang-Ming University).

### 2.2. Preparation of Dispo85E

Dried and peeled tubers of *Dioscorea alata* L. cv. Phyto (2.92 kg) were extracted with 85% ethanol (EtOH) (15 L each, three times) for 24 hours. The extract was filtered through an 11 *μ*m Whatman filter, then concentrated under vacuum evaporator and lyophilized, giving a yield of 3.05%. The extract was stored at −20°C before use. 

### 2.3. HPLC Analysis of Dispo85E

Twenty milligrams of dried Dispo85E was dissolved in 2 mL of dichloromethane (DCM). After centrifugation, the supernatant was evaporated to dryness, adjusted to a concentration of 5 mg/mL in DCM and then subjected to normal-phase high performance liquid chromatography (NP-HPLC) with a 100 *μ*L injection volume. The pellet was evaporated to dryness, adjusted to a concentration of 10 mg/mL in water and then subjected to reverse-phase high performance liquid chromatography (RP-HPLC) with a 100 *μ*L injection volume. RP-HPLC profiling was performed using a Mightysil RP-18 column (4.6 × 250 mm, 5 mm) at room temperature. The mobile phase was methanol and water in gradient mode (10 : 90–100 : 0 over 120 minutes). The effluent was monitored at 254 nm, and a constant flow rate was set at 0.8 mL/minute. NP-HPLC profiling was performed with a Cosmosil 5SL-II column (4.6 × 250 mm, 5 mm) at room temperature. The mobile phase was DCM and methanol (MeOH) in gradient mode as follows: 100%–98% DCM in MeOH (0–15 minutes), 98%-95% DCM (15–25 minutes), 95%–90% DCM (25–35 minutes), 90%–80% DCM (35–45 minutes), and 80%–70% DCM (45–55 minutes). The effluent was monitored at 254 nm, and a constant flow rate was set at 0.8 mL/minute.

### 2.4. Cell Culture

Primary mouse bone marrow cells and C3H10T1/2 cells were cultured as described previously [12, 13]. Briefly, bone marrow cells were obtained from the femoral bone of SPF-grade C57BL/6 female mice. The bone marrow cells were collected by flushing the diaphysis with *α*-minimum essential medium (*α*-MEM, Gibco BRL) through a 23-gauge needle. After flushing, the bone marrow cells were filtered through a no. 53 sterile nylon mesh to obtain a single cell suspension. The cells were cultured in osteogenic medium (*α*-MEM medium supplemented with 15% fetal calf serum (FCS, Gibco), 50 *μ*g/mL ascorbic acid (Sigma), 10 mm sodium *β*-glycerophosphate (Sigma), and 10 nm dexamethasone (Sigma)). The pluripotent murine mesenchymal cell line C3H10T1/2 was cultured in Dulbecco's modified Eagle's medium (DMEM) supplemented with 10% FCS.

For the alkaline phosphatase (ALP) activity assay, primary bone marrow cells were seeded in a 96-well microplate at 2.5 × 10^5^/well and cultured in osteogenic induction medium. After two days, half of the medium was changed and the cells were incubated with Dispo85E at the indicated concentrations for 4 days. ALP activity for each cell lysate was assayed. C3H10T1/2 cells were grown to confluence in standard medium. One day after the cells reached confluence, the medium was replaced with permissive osteogenic/adipocytic medium [14] (DMEM with 10% FCS, 50 *μ*g/mL ascorbic acid, 10 *μ*M sodium *β*-glycerophosphate, 10 nm dexamethasone, 10 nm all-trans retinoic acid (Sigma), 10 *μ*g/mL insulin (Sigma), and 50 *μ*M isobutylmethylxanthine (IBMX, Sigma)) containing drugs at the indicated concentrations. The medium was changed every 4 days. After 12 days, ALP activity for each cell lysate was assayed.

For the nodule formation assay, primary bone marrow cells were seeded in a 24-well microplate at 1 × 10^6^/well and cultured in osteogenic induction medium. After two days, half of the medium was changed and the cells were incubated with Dispo85E at the indicated concentrations. The culture medium was renewed every 4 days. After 14 days, the mineralization of bone marrow cells was analyzed.

For lipid staining, C3H10T1/2 cells were grown to confluence in standard medium. One day after the cells reached confluence, the medium was replaced with adipogenic medium (DMEM with 10% FCS, 10 *μ*g/mL insulin, 1 *μ*M dexamethasone, and 0.5 mm IBMX) containing drugs at the indicated concentrations for 4 days. The cells were then incubated in a standard medium containing 5 *μ*g/mL insulin and drugs at the indicated concentrations. The medium was changed every 4 days. The oil red O staining was performed at postinduction day (PID) 12. Nile red staining was performed at PID16.

### 2.5. Alkaline Phosphatase Activity Assay

ALP activity was assayed at 37°C by a method modified from that of Qu et al. [15]. In brief, cell layers were washed twice with phosphate buffered saline (PBS) and extracted into lysis solution (10 mm Tris, 0.1% Triton X-100 buffer (pH 7.5)). Enzyme activity was determined colorimetrically using p-nitrophenylphosphate (p-NPP, Sigma) as a substrate. The reaction mixture contained 8 mm p-NPP, 2 mm mgCl_2_, and 0.5 M 2-amino-2-methyl-1-propanol, pH 10. After 10 minutes of incubation, the color change of p-NPP to p-nitrophenol was monitored at 405 nm. Cell viability was determined by resazurin assay. Fifty micromolar resazurin (Sigma) was added to the cell culture medium and incubated for 2 hours. Medium samples were collected into a 96-well plate, and fluorescence was measured with excitation at 544 nm and emission at 590 nm.

### 2.6. Nodule Formation Assay

The *in vitro* mineralized nodule formation assay was performed as described previously [16]. In brief, cells were fixed with 10% formalin for 30 minutes at 37°C. The formalin was removed, and the cells rinsed with sterilized water three times. Next, 2% alizarin red S solution, which reacts with calcium, was added to the wells and the cells were incubated at 37°C for 10 minutes. The Alizarin solution was removed, and the cells were washed with water and dried in air. Stained cultures were photographed, and calcium deposition was quantified by extracting alizarin red S staining with 10% cetylpyridinium chloride (CPC, Sigma) and measuring the OD of the extract at 550 nm [17].

### 2.7. Adipocyte Staining with Oil Red O

After differentiation was induced, cells were stained with oil red O. Briefly, cells were washed twice with PBS and fixed with 10% formalin in PBS for 1 hour. They were then washed three times with water. Cells were stained with Oil Red O (six parts of 0.6% oil red O dye (Sigma-Aldrich) in isopropanol and four parts of water) for 30 minutes. Excess stain was removed by washing with water, and the stained cells were dried. Adipocytes stained red were observed under the phase contrast microscope with 200-fold magnification.

### 2.8. Quantification of Adipocyte Number by Flow Cytometry

The formation of mature adipocytes was quantified with flow cytometry using the lipophilic Nile red fluorescent dye as described previously [18]. Briefly, after the cells were induced to differentiate into adipocytes as indicated above, they were washed twice in PBS, trypsinized, pelleted by centrifugation, and fixed in 10% formaldehyde for 1 h at 4°C. A working solution of Nile red was prepared by dissolving 1 mg Nile red in 200 *μ*L DMSO and diluting to 100 mL with PBS. The cells were stained with 10 *μ*g/mL Nile red for 45 minutes at room temperature. The fluorescent emission was detected between 564 nm and 604 nm with a band-pass filter using a FACScan flow cytometer (BD Biosciences) linked with Cell-Quest 3.3 software (BD Biosciences).

### 2.9. Isolation and Analysis of RNA

Total RNA was extracted from the bone marrow cells using the Ultraspec RNA isolation kit (Biotex laboratories INC, USA). RNA was reverse transcribed using AMV reverse transcriptase (Promega, USA) and an appropriate buffer in a final reaction volume of 40 *μ*L. The complementary DNA (cDNA) was obtained using the previous reaction solution at 42°C for 60 minutes, followed by 90°C for 5 minutes. The resultant cDNA (2.5 *μ*L) was added to 0.5 *μ*L 10 mm dNTP, 0.5 *μ*L polymerase (2 units), and 1 *μ*L of 10 *μ*M the appropriate primers, and the final volume of the reaction mixture was adjusted to 25 *μ*L. Polymerase chain reaction (PCR) was performed for 35 cycles with each cycle consisting of 45 seconds of denaturation at 94°C, 45 seconds of annealing at proper annealing temperature, and 1 minute of extension at 72°C. The primers used were as follows: OCN (5′-TCT  GAC  AAA  GCC  TTC  ATG  TCC-3′ and 5′-AAA  TAG  TGA  TAC  CGT  AGA  TGC  G-3′), IGF-1 (5′-GCT  CTT  CAG  TTC  GTG  TGT  GG-3′ and 5′-TTG  GGC  ATG  TCA  GTG  TGG-3′), BMP-2 (5′-CAT  CCA  GCC  GAC  CCT  TG-3′ and 5′-CTC  TCC  CAC  TGA  CTT  GTG-3′), ALP (5′-GCC  CTC  TCC  AAG  ACA  TAT  A-3′ and 5′-CCA  TGA  TCA  CGT  CGA  TAT  CC-3′), COL-*Ⅰ* (5′-TCT  CCA  CTC  TTC  TAG  TTC  CT-3′ and 5′-TTG  GGT  CAT  TTC  CAC  ATG  C-3′), *β*-actin (5′-GAC  TAC  CTC  ATG  AAG  ATC  CT-3′ and 5′-CCA  CAT  CTG  CTG  GAA  GGT  GG-3′), IL-4 (5′-TG  GGT  CTC  AAC  CCC  CAG  CTA  GT-3′ and 5′-GCT  CTT  TAG  GCT  TTC  CAG  GAA  GTC-3′), IL-6 (5′-ATG  AAG  TTC  CTC  TCT  GCA  AGA  GAC  T-3′ and 5′-CAC  TAG  GTT  TGC  CGA  GTA  GAT  CTC-3′), and TGF-*β* (5′-TGG  ACC  GCA  ACA  ACG  CCA  TCT  ATG  CCA  TCT  ATG  AGA  AAA  CC-3′ and 5′-TGG  AGC  TGA  AGC  AAT  AGT  TGG  TAT  CCA  GGG  CT-3′). The reaction products were analyzed by electrophoresis on a 2% agarose gel and visualized by ethidium bromide staining with ultraviolet light illumination. Gene expression levels were analyzed by IMAGEQUANTE software and normalized with *β*-actin. Real-time PCR was performed using an Applied Biosystems 7500 Real-Time PCR System (Applied Biosystems). The PCR mixture was prepared using SYBR green Mastermix (Roche). The thermal cycling conditions were 10 minutes at 95°C followed by 40 cycles of 95°C for 15 seconds and 57°C (OCN and IGF-1) for 20 seconds, with an extension period at 72°C for 40 seconds. Expression of each gene was normalized to GAPDH mRNA content.

### 2.10. Animal Models

There were two different sets for the *in vivo* experiment. In the first series of experiments, intact female mice (without OVX surgery) were fed a normal diet containing different concentrations (0, 40, 200, and 1000 mg/kg/day) of Dispo85E *ad libitum* for 5 days (*n* = 4 for each group). In the second set of experiments, we used an OVX mouse model to mimic osteoporosis in postmenopausal women. The mice were subjected to surgery at day 0. Sham surgery was performed by identifying the bilateral ovaries, and OVX was performed by removing the bilateral ovaries. Mice (*n* = 10-11 for each group) were initially fed a normal diet containing different concentrations (0, 40, 200, and 1000 mg/kg/day) of Dispo85E *ad libitum*. Feeding began soon after OVX surgery and continued for 42 days. We identified OVX mice by confirming uterus atrophy in OVX animals at the end of the experiment. At the end of the experiment, all mice were sacrificed by cervical dislocation. Bone marrow cells were then acquired from the femur of each mouse. The drug's effect on ALP activity, nodule formation, mRNA expression of the osteoblast differentiation related genes in bone marrow cells, histological change of the trabecular bone, trabecular bone mineral density (tBMD), and bone tissue microarchitecture were analyzed. To analyze the drug's effect on the gene expression of bone marrow cells, we extracted total RNA from bone marrow cells of the femora of the intact or OVX mice after continuous feeding with Dispo85E and examined expression through RT-PCR. To analyze the drug's effect on the ALP activity of bone-marrow-derived cells isolated from intact or OVX mice, the mice were sacrificed to isolate the bone marrow cells after treatment with different doses of Dispo85E. The isolated bone marrow cells were seeded in a 96-well microplate at 2.5 × 10^5^/well and cultured in osteogenic induction medium for 6 days without additional Dispo85E treatment. After 6 days of culture, the ALP activity for each cell lysate was assayed.

### 2.11. Histological Analysis of Trabecular Bone

Histomorphometric measurements of trabecular bone volume were performed as described previously with minor modifications [12]. In brief, femora were collected 42 days after the surgical operation, fixed in Bouin's solution at 26°C for 24 hours, decalcified in 14% EDTA, dehydrated in progressive concentrations of ethanol, cleared in xylene, and embedded in paraffin. Five-micron-thick sections were cut and stained for hematoxylin and eosin Y. Static parameters were measured in the square, distal metaphysics of the femur within the endosteal surfaces, excluding the epiphyseal growth plate, and 1 mm distal from the end of the calcified cartilage. Histomorphometric measurements of the bone volume/total bone volume percentage (BV/TV, %) were performed at 40-fold magnification.

### 2.12. Analysis of Trabecular Bone Microarchitecture

The OVX mice were sacrificed 42 days after beginning the treatment with Dispo85E. The femurs were aseptically removed, cleansed of adherent soft tissues, and deposited in a tube with 10% formalin. The trabecular bone microarchitecture of the distal femoral metaphysis was scanned by microcomputed tomography (*μ*CT, Skyscan 1076) in the region of 0.6–2.1 mm from the growth plate. The X-ray source was set at a voltage of 48 kV and a current of 200 *μ*A and filtered with a 0.5 mm aluminum filter. The scanning angular rotation was 180° with an angular step of 0.7°. The voxel size was isotropic and fixed at 8.7 *μ*m. The trabecular bone parameters were calculated using the Skyscan software CTan (Skyscan). Morphometric indices of the trabecular bone region were determined from the microtomographic data sets using direct 3D morphometry. Trabecular bone volume (BV/TV; %), trabecular thickness (Tb.Th; mm), and trabecular number (Tb.N; 1/mm) were calculated. The volumetric trabecular bone mineral density (tBMD) was also determined by *μ*CT scanning. tBMD was calculated in the conforming volume of interest described previously for the trabecular region.

### 2.13. Statistical Analysis

All results are expressed as the mean and standard deviation (SD). The statistical significance was evaluated by one-way analysis of variance (ANOVA) followed by Dunnett's test. A level of *P* < .05 was considered statistically significant. 

## 3. Results

### 3.1. HPLC Analysis of Dispo85E

**Figure 1:**
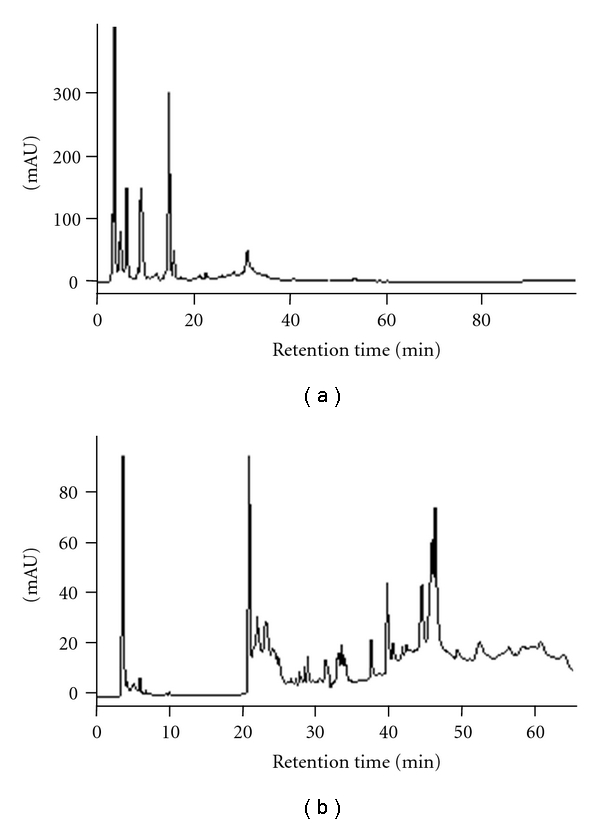
HPLC analysis of Dispo85E. (a) Reverse-phase HPLC profile. Column: Mightysil RP-18 (4.6 × 250 mm, 5 mm); mobile phase: methanol-water (methanol: 10–100% in 120 minutes); flow rate: 0.8 mL/minute; detection wavelength: 254 nm. (b) Normal-phase HPLC profile. Column: Cosmosil 5SL-II (4.6 × 250 mm, 5 mm); mobile phase: 100%–98% DCM in MeOH (0–15 minutes), 98%–95% DCM (15–25 minutes), 95%–90% DCM (25–35 minutes), 90%–80% DCM (35–45 minutes), and 80%–70% DCM (45–55 minutes); flow rate: 0.8 mL/minute; detection wavelength: 254 nm.

Dispo85E is a mixture extracted from the traditional botanical *Dioscorea alata* L. cv. Phyto. This mixture contains both polar and nonpolar small molecule ingredients. The-reverse phase HPLC analysis showed the polar to semipolar ingredient fingerprint of Dispo85E, and the normal phase HPLC analysis showed the nonpolar ingredient fingerprint of Dispo85E (Figure 1).

### 3.2. Dispo85E Promotes Differentiation of Osteoblastic Cells in Bone Marrow Culture

**Figure 2:**
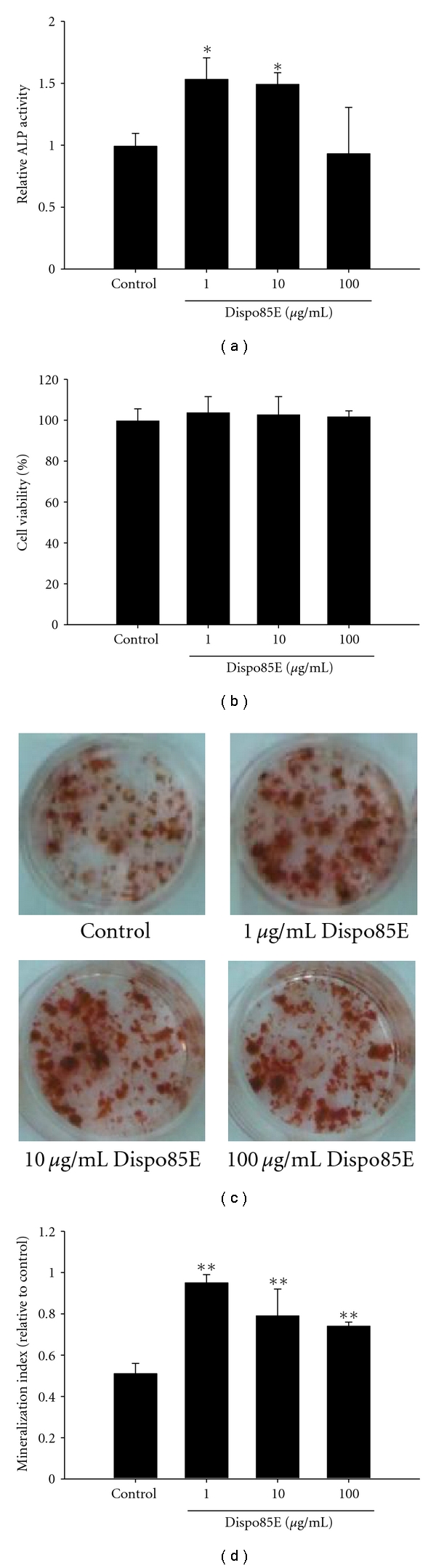
Dispo85E stimulates osteoblastogenesis in bone-marrow-derived cells. Primary bone-marrow-derived cells were cultured with osteogenic medium and treated with Dispo85E at the indicated concentrations. At PID 6, (a) cellular alkaline phosphatase (ALP) activity and (b) the effect of Dispo85E on cell viability were measured (*n* = 4). (c) At PID 16, cells were stained with alizarin red S, and (d) the mineralized area was quantified by extracting alizarin red S staining with 10% cetylpyridinium chloride (CPC) and measuring the OD of the extract at 550 nm (*n* = 3). **Indicates significant difference from control group at *P* < .01; *Indicates significance at *P* < .05.

The ALP activity assay showed that bone marrow cells treated with Dispo85E had a 1.5-fold maximal increase in the activity of ALP (Figure 2(a)). In the cell viability assay, there were no significant differences between the control and Dispo85E-treated groups (Figure 2(b)). Furthermore, Dispo85E significantly stimulated mineralization of bone marrow cultures by alizarin red staining (Figures 2(c) and 2(d)).

### 3.3. Dispo85E Promotes Osteoblastogenesis and Inhibits Adipogenesis in the Mesenchymal Stem Cell Line C3H10T1/2

**Figure 3:**
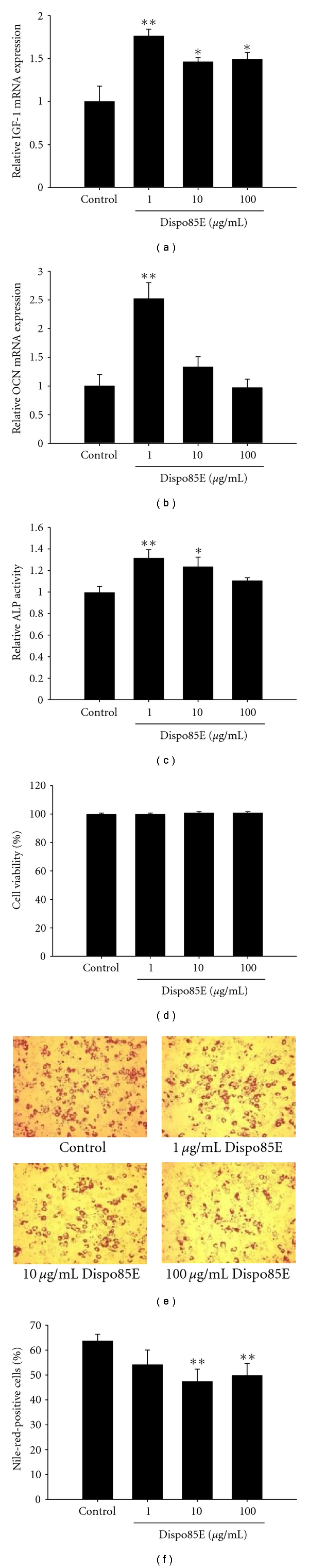
Dispo85E stimulates osteoblastogenesis and inhibits adipogenesis in the mesenchymal stem cell line C3H10T1/2. C3H10T1/2 cells were grown to confluence in a standard medium. One day after confluence, the medium was replaced with a permissive osteogenic/adipogenic medium containing Dispo85E at the indicated concentrations. At PID 12, the mRNA level of IGF-1 (a) and OCN (b) was analyzed by real-time PCR (*n* = 3). (c) ALP activity and (d) the effect of Dispo85E on cell viability were measured (*n* = 4). (e) Cells were induced with adipogenic medium and treated with drugs. At PID 12, the cells were stained with oil red O to assess lipid accumulation (magnification, 200-fold). (f) After 16 days of culture in adipogenic medium, cells were harvested, fixed, and stained with Nile red solution. Percentage of Nile-red-stained cells in the total population of each sample were quantified with FACScan flow cytometry (*n* = 3). **Indicates significant difference from control group at *P* < .01; *Indicates significance at *P* < .05.

The real-time PCR results showed that mRNA expression of IGF-1, which could promote osteoblast differentiation and bone formation, was significantly increased after treatment with Dispo85E (Figure 3(a)). OCN (an osteoblastic marker) mRNA expression was significantly increased by approximately 2.5-fold after treatment with 1 *μ*g/mL Dispo85E (Figure 3(b)). Concomitantly, we observed increased ALP activity in C3H10T1/2 cells treated with Dispo85E (Figure 3(c)), which had no effect on cell viability (Figure 3(d)). 

The effects of Dispo85E on adipocyte differentiation were detected by oil red O staining (Figure 3(e)) and quantified by Nile red staining and flow cytometry (FACS) analysis (Figure 3(f)). After 12 days in adipogenic medium, C3H10T1/2 cells showed an abundance of oil-red-O-stained lipid droplets (Figure 3(e)). Dispo85E resulted in a dose-dependent decrease in cellular lipid accumulation (Figure 3(e)). Using flow cytometry and Nile red staining, the percentage of adipocytes were calculated based on the number of stained cells. After culture in adipogenic medium for 16 days, 63.6% of the cells were calculated to be adipocytes (Figure 3(f)). After treatment with 1, 10, or 100 *μ*g/mL Dispo85E, the percentages of adipocytes were 54.1%, 47.3%, and 49.8%, respectively (Figure 3(f)).

### 3.4. Dispo85E Promotes Osteoblastogenesis in Intact Mice (without OVX Surgery)

**Gene Expression**

**Figure 4:**
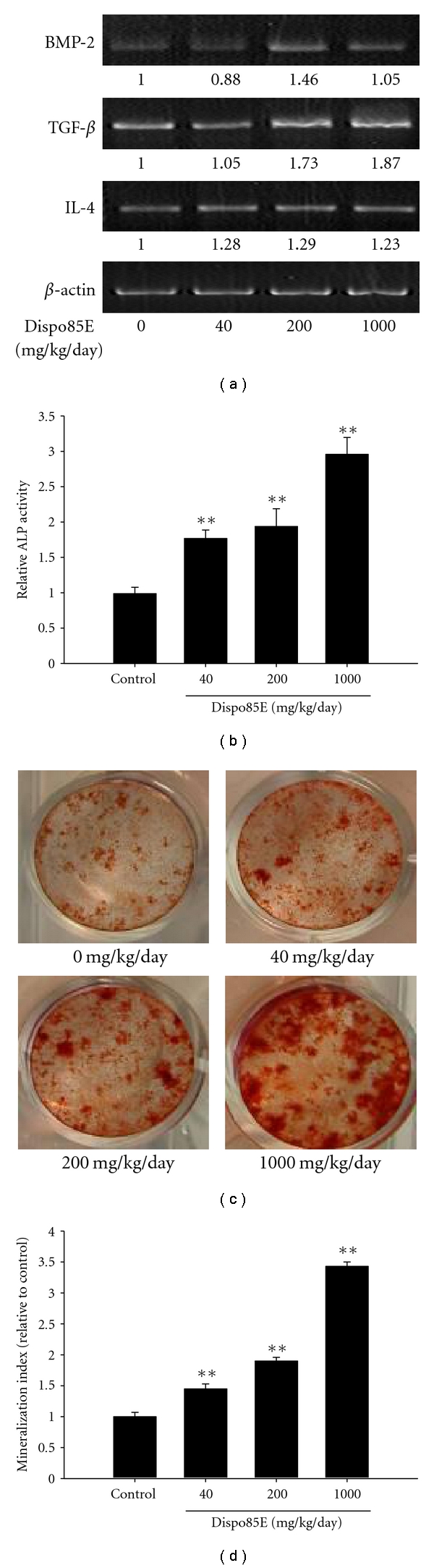
Dispo85E stimulates osteoblastogenesis in bone-marrow-derived cells isolated from intact mice. C57BL/6 mice were fed with 0 mg/kg/day (vehicle control), 40 mg/kg/day, 200 mg/kg/day, or 1000 mg/kg/day Dispo85E for 5 days. (a) Expression of mRNA for different bone regulatory factors in mouse bone-marrow-derived cells was evaluated by RT-PCR. The amplified fragments were visualized on ethidium-bromide-stained agarose gels. (b) Cellular ALP activity and (c) nodule formation assay were performed as described in Section 2. Cells were stained with alizarin red S for osteoblasts and (d) the mineralized area was quantified using Meta Image software. **Indicates significant difference from control group at *P* < .01. *n* = 4 for each group.

We used the expression level of *β*-actin mRNA as a house-keeping gene. Compared to the expression of *β*-actin mRNA, the relative levels of BMP-2, TGF-*β*, and IL-4 mRNA were higher in bone marrow cells from Dispo85E-treated mice than those from untreated controls (Figure 4(a)).

**Alkaline Phosphate Activity Assay**

We measured the specific activity of ALP of bone marrow cells isolated from Dispo85E-fed or control mice for 5 days. The ALP activity was significantly higher in all Dispo85E-treated groups (Figure 4(b)). Dispo85E at a dose of 1000 mg/kg created up to a 3-fold enhancement compared to the control group.

**Nodule Formation Assay**

To evaluate whether Dispo85E enhanced bone mineralization, we used alizarin red staining to visualize nodule formation in bone marrow cultures (Figure 4(c)). The proportional area of alizarin-red-positive staining in the Dispo85E-treated group was larger than that in the untreated group in a dose-dependent manner (Figure 4(d)). Compared with the control group, 1000 mg/kg Dispo85E showed the strongest enhancement, with effects up to 3.4-fold above controls (Figure 4(d)).

### 3.5. Dispo85E Promotes Osteoblastogenesis in OVX-Induced Osteoporotic Mice

**Gene Expression**

**Figure 5:**
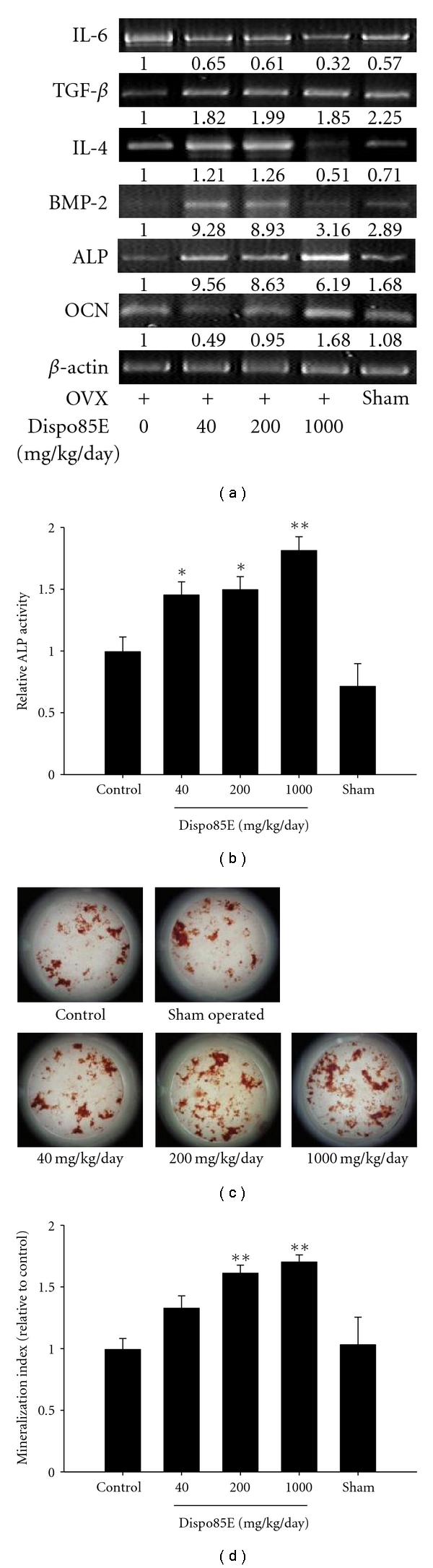
Dispo85E stimulates osteoblastogenesis in bone-marrow-derived cells isolated from OVX mice. C57BL/6 mice were sham or OVX. The OVX mice were fed 0 mg/kg/day (vehicle control), 40 mg/kg/day, 200 mg/kg/day, or 1000 mg/kg/day Dispo85E for 42 days. (a) Expression of the mRNA for different bone regulatory factors and osteoblastic markers in OVX mouse bone-marrow-derived cells was evaluated by RT-PCR. (b) Cellular ALP activity and (c) nodule formation assay were performed as described in Section 2. Cells were stained with Alizarin red S for osteoblasts, and (d) the mineralized area was quantified by extracting alizarin red S staining with 10% CPC and measuring the OD of the extract at 550 nm. **Indicates significant difference from OVX mice at *P* < .01; *Indicates significance at *P* < .05. *n* =10-11 for each group.

Bone morphogenetic protein-2 (BMP-2), transforming growth factor-*β* (TGF-*β*), and interleukin-4 (IL-4) are positively related to the proliferation or differentiation of bone marrow cells toward an osteoblast lineage. ALP, collagen I (COL-I), and osteocalcin (OCN) are widely used as bone formation or osteoblastic cells markers. Interleukin-6 (IL-6) is a bone resorption marker. When compared to *β*-actin, the relative levels of BMP-2, TGF-*β*, IL-4, ALP, COL-I, and OCN mRNA were higher in bone marrow cells from Dispo85E-treated mice than in untreated mice. Conversely, the bone resorption marker gene IL-6 was decreased after treatment with Dispo85E (Figure 5(a)).

**Alkaline Phosphate Activity Assay**

The ALP activity of all Dispo85E-treated groups was significantly higher than that of the untreated group (Figure 5(b)).

**Nodule Formation Assay**

Groups treated with 200 and 1000 mg/kg/day Dispo85E showed enhanced bone mass mineralization compared to the untreated group (Figures 5(c) and 5(d)).

**Histological Analysis of Trabecular Bone**

**Figure 6:**
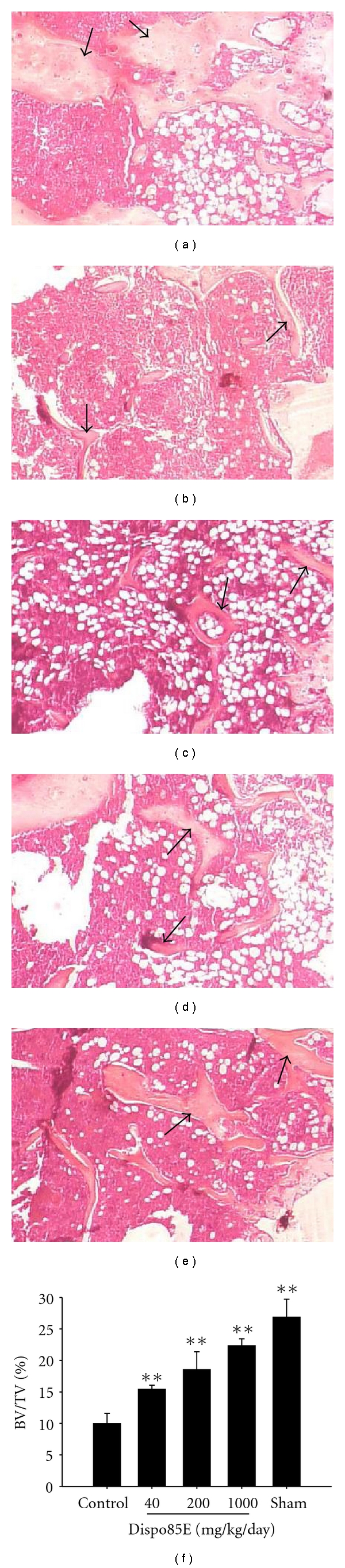
Effect of Dispo85E on trabecular bone volume in OVX mouse model. C57BL/6 mice were sham operated (a) or OVX. The OVX mice were fed 0 mg/kg/day (vehicle control) (b), 40 mg/kg/day (c), 200 mg/kg/day (d), or 1000 mg/kg/day (e) Dispo85E for 42 days. The femora were collected 42 days after the operation, and the sections of distal femoral metaphysis were stained with Hematoxylin-eosin. Arrow indicates the trabecular bone. (f) Histomorphometric measurements of bone volume/total bone volume percentage (BV/TV, %) were performed. Measurement was performed at a magnification of 40-fold. **Indicates significant difference from OVX mice at *P* < .01. *n* = 5 for each group.

Histological analysis of the trabecular bone was performed in the OVX mouse model. The animal data in the sham-operated positive control group showed a significant increase in the trabecular bone volume/total bone volume percentage (BV/TV, %) compared to the vehicle control group. There was a significant increase in BV/TV for all dosage groups compared to the vehicle control group (0 mg/kg/day). Moreover, the increase was positively correlated with dose level. The histomorphometric analysis in the OVX model revealed increases in the osteoid volume in a dose-dependent manner (Figure 6), suggesting that Dispo85E ameliorates the decreasing trabeculation of the bone marrow spaces in osteoporotic mice.

**Analysis of Trabecular Bone Microarchitecture**

The effects of Dispo85E on trabecular bone microarchitecture and trabecular BMD of the distal femur in OVX mice were assessed by *ex vivo μ*CT analysis. Quantification of the trabecular bone changes in the distal femoral metaphysis is shown in Table 1. Compared with the sham-operated animals, the OVX control group showed a significant decrease in trabecular bone mineral density (tBMD), trabecular bone volume fraction (BV/TV), and trabecular number (Tb.N). Both 40 mg/kg/day and 1000 mg/kg/day of Dispo85E significantly improved various *μ*CT bone parameters, including tBMD, BV/TV, and Tb.N compared to the OVX control group, but trabecular thickness (Tb.Th) was not significantly altered compared to the OVX control (Table 1).

## 4. Discussion

Several drugs have been reported to be effective in treating osteoporosis. However, most of these drugs are inhibitors of osteoclast-mediated bone resorption and present many detrimental side effects [19]. For example, estrogen replacement therapy has been demonstrated to prevent bone loss in postmenopausal women [20]. However, despite the benefits of estrogen, many studies have also shown that estrogen would increase the incidence of ovarian cancer and breast cancer [7, 8]. Furthermore, epidemiological reports have shown that hormone replacement therapy (HRT) is associated with increases in the incidence rates of breast cancer, heart attacks, stroke, and blood clot formation. Such studies have prompted the US FDA to issue safety warnings and to suggest the avoidance of HRT as a general preventative osteoporosis therapy [20, 21]. On the other hand, Teitelbaum highlighted that compounds that can stimulate new bone formation may offer safe and clinically effective prevention and reversal of osteoporosis [22].

Bone marrow cells have long been recognized as the source of osteoprogenitor cells [23]. Thus, we applied a culture model of primary bone marrow cells to evaluate the effect of Dispo85E on the differentiation of osteoblasts. Intriguingly, our observations of ALP activity and mineralization suggested that Dispo85E possessed higher efficacy in promoting osteoblast differentiation. In this assay, we measured cell viability and found no significant difference between the control and treated groups. Therefore, we suggest that Dispo85E does not influence the proliferation of stromal cells *in vitro*.

Recently, it has been suggested that osteoporosis is caused by an increased ratio of adipocytes to osteoblasts and not by an imbalance between osteoclasts and osteoblasts alone [24]. Indeed, an increase in adipogenesis in bone marrow associated with osteoporosis is well known clinically [25]. Other studies have also demonstrated that increases in the number of marrow adipocytes caused bone loss in osteoporosis animal models, such as in ovariectomized or glucocorticoid-treated animals [26, 27]. It was reported that the number of osteoblasts correlated negatively with the number of adipocytes, which suggested that an inverse relationship between osteogenic and adipogenic differentiation exists [28, 29].

It is known that multipotent mesenchymal stem cells (MSCs) from bone marrow stroma can differentiate into various cells, including osteoblasts and adipocytes [30, 31]. A shift in differentiation and survival rates from an osteoblastic to adipocytic lineage can lead to an altered ratio of fat to bone cells and, ultimately, an alteration in bone mass [31]. Thus, it has been suggested that targeting regulatory factors to direct the fate of mesenchymal cells toward osteoblasts may provide novel therapeutic approaches for osteoporosis [32]. In this study, we used the C3H10T1/2 cell line, which maintains the differentiating characteristics of mesenchymal stem cells [33], to evaluate the effect of Dispo85E on the osteogenesis/adipogenesis balance. Our *in vitro* data showed that Dispo85E could promote the differentiation of mesenchymal stem cells into the osteoblastic lineage and reduce adipogenic differentiation. Recently, it has been reported that the number of adipocytes in bone marrow correlated inversely with the hematopoietic activity of the marrow [34]. We also suggest that the antiadipogenic effect of Dispo85E might have the potential for use in some hematopoietic disorders.

Insulin-like growth factor I (IGF-I) is known as an important factor produced by osteoblasts to regulate bone formation and remodeling [35]. It can induce human adult mesenchymal stem cells (hMSCs) to differentiate into an osteogenic lineage [36]. Some clinical data show that IGF-I was lower in women with osteoporosis than in women with normal bone mineral density (BMD) [37]. In addition, low serum IGF-1 can contribute to reduced BMD, reduced osteoblast differentiation, and increased the numbers of adipocytes in bone [38]. Real-time PCR showed that Dispo85E increased IGF-1 mRNA expression in C3H10T1/2 cells. Thus, we suggest that one way Dispo85E regulates the differentiation of MSCs is through IGF-1 upregulation.

To evaluate whether Dispo85E enhanced bone formation *in vivo*, we used two kinds of animal models. In the intact mouse model, we evaluated the ability of Dispo85E to promote osteoblastogenesis under physiological conditions. PCR showed that Dispo85E increased BMP-2, TGF-*β*, and IL-4 gene expression in bone marrow cells. BMP-2 has been known to stimulate osteoblastic maturation and collagen synthesis in osteoblastic cells [39], TGF-*β* has been reported to stimulate the proliferation of osteoblast precursors and increase the pool of committed osteoblasts [40], and IL-4 is a potent inhibitor of bone resorption [41]. In addition, the results of the ALP activity assay and alizarin red staining for mineralization of bone marrow showed that Dispo85E promotes osteoblast differentiation in healthy animals.

Next, we used an OVX mouse model to mimic the osteoporosis seen in postmenopausal women. After 6 weeks of treatment, Dispo85E promoted osteoblastogenesis without a significant effect on uterine weight (data not shown). This result differs from the previously observed restorative effects of estrogen replacement [42]. Therefore, we suggest that Dispo85E might act via a different mechanism from estrogen, which affects the uterus as well as bone. In this animal study, we analyzed bone marrow cells after 42 days of OVX. Our results showed that ALP activity in bone marrow cells (cultured in osteogenic induction medium for 6 days without additional Dispo85E treatment) of Dispo85E-treated groups was higher than that of the OVX control group. These data indicated that Dispo85E-treated groups contained more osteoprogenitor cells than the untreated group in the OVX mice model.

Previous studies have clearly shown a reduction of trabecular bone volume during aging and in osteoporosis patients [43]. In our study, histological analysis of the femora harvested from OVX mice showed an empty bone marrow space in the trabecular bone. After administration of Dispo85E for 42 days, the trabecular bone volume was increased. Additionally, the *μ*CT data confirmed that 40 mg/kg/day and 1000 mg/kg/day of Dispo85E could significantly ameliorate the deterioration of both tBMD and trabecular microarchitectural parameters in OVX mice. The 200 mg/kg/day group also showed better indices than the untreated group, although it did not reach statistical significance. This may be due to the limited number of animals in our study or inconsistencies in the mixture chemistry of the Dispo85E extract. These data indicated that Dispo85E promotes osteoblast differentiation and bone formation in an osteopenic setting without side effects on the uterus.

Recent studies have shown that natural products have potential as alternative approaches to prevent and ameliorate osteoporosis through different mechanisms. For example, Xie et al. reported that *Herba epimedii* (HEP) extract can effectively suppress the OVX-induced increase in bone turnover, possibly by both an increase in osteoblastic activities and a decrease in osteoclastogenesis [44]. Ho et al. reported that *Flemingia macrophylla* extract can ameliorate experimental osteoporosis in OVX rats through inhibition of bone resorption [45]. Hidaka et al. reported that royal jelly (RJ) can prevent osteoporosis by enhancing intestinal calcium absorption [46]. In these studies, most of the drugs have been reported to achieve improvement in osteoporosis by regulating the balance between osteoblasts and osteoclasts. Unlike these drugs, our study has shown that Dispo85E can drive the lineage-specific differentiation of bone marrow stromal cells to differentiate into osteoblasts rather than adipocytes. Furthermore, our data showed that Dispo85E could promote the differentiation and maturation of osteoblasts in both intact and OVX mice. Therefore, we suggest that Dispo85E not only can decrease the process of bone loss but also can regulate the balance between osteoblasts and adipocytes to promote bone formation. 

**Figure 7:**
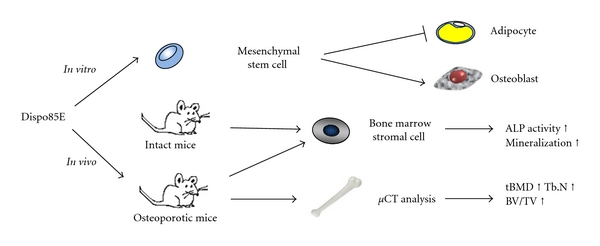
Dispo85E promotes bone formation by driving differentiation of mesenchymal stem cells into osteoblasts rather than adipocytes and ameliorates osteoporosis in the mouse model.

Based on these findings, we conclude that Dispo85E regulates mesenchymal stem cell differentiation into an osteogenic lineage rather than an adipogenic lineage and ameliorates osteoporosis in a mouse model (Figure 7). Our data suggest that Dispo85E induces mesenchymal stem cell differentiation and holds promise as a novel therapeutic drug for the treatment of osteoporosis.

## Figures and Tables

**Table 1:** Effects of Dispo85E on tBMD and morphometric parameters of distal femur in OVX model.

Parameters	Sham	OVX + Dispo85E (mg/kg/day)			
		0	40	200	1000
tBMD (g/cm^3^)	0.088 ± 0.003	0.054 ± 0.008*	0.083 ± 0.003^‡^	0.063 ± 0.009*	0.081 ± 0.006^‡^
BV/TV (%)	4.34 ± 0.36	2.56 ± 0.44*	4.17 ± 0.29^†^	3.50 ± 0.69	4.11 ± 0.39^†^
Tb.Th (*μ*m)	60.7 ± 1.03	59.30 ± 0.71	64.36 ± 2.25	63.15 ± 2.70	63.03 ± 2.26
Tb.N (1/mm)	0.72 ± 0.06	0.43 ± 0.07*	0.65 ± 0.04^†^	0.55 ± 0.10	0.66 ± 0.06^†^

Values are expressed as mean ± SD; *n* = 5-6/group. tBMD: trabecular bone mineral density; BV/TV: bone volume fraction; Tb.Th: trabecular thickness; Tb.N: trabecular number.

*P < .05 compared with the sham operated group;

^†^ *P* < .05 compared with OVX control group;

^‡^ *P* < .01 compared with OVX control group.
